# Healthy ageing has divergent effects on verbal and non-verbal semantic cognition

**DOI:** 10.1177/17470218231195341

**Published:** 2023-08-23

**Authors:** Wei Wu, Suchismita Lohani, Taylore Homan, Katya Krieger-Redwood, Paul Hoffman

**Affiliations:** 1 School of Philosophy, Psychology & Language Sciences, The University of Edinburgh, Edinburgh, UK; 2 Department of Psychology, University of York, York, UK

**Keywords:** Semantic cognition, cognitive ageing, semantic knowledge, semantic control, modality

## Abstract

Semantic cognition refers to the storage and appropriate use of knowledge acquired over the lifespan and underpins our everyday verbal and non-verbal behaviours. Successful semantic cognition requires representation of knowledge and control processes which ensure that currently relevant aspects of knowledge are retrieved and selected. Although these abilities have been widely studied in healthy young populations and semantically impaired patients, it is unclear how they change as a function of healthy ageing, especially for non-verbal semantic processing. Here, we addressed this issue by comparing the performance profiles of young and older people on a semantic knowledge task and a semantic control task, across verbal (word) and non-verbal (picture) versions. The results revealed distinct patterns of change during adulthood for semantic knowledge and semantic control. Older people performed better in both verbal and non-verbal knowledge tasks than young people. However, although the older group showed preserved controlled retrieval for verbal semantics, they demonstrated a specific impairment for non-verbal semantic control. These findings indicate that the effects of ageing on semantic cognition are more complex than previously assumed, and that input modality plays an important role in the shifting cognitive architecture of semantics in later life.

## Introduction

Semantic cognition is a fundamental ability of humans, bringing meaning to our memories and ongoing experiences and allows us to use our knowledge to drive context-appropriate behaviour (Badre & Wagner, 2002; Hoffman et al., 2018; Jefferies, 2013; Lambon Ralph et al., 2017). Effective semantic cognition is thought to be supported by two key components: semantic knowledge representations and semantic control processes (Jefferies, 2013; Lambon Ralph et al., 2017). Representation of semantic knowledge involves storage of a wealth of information about the complex world, while semantic control processes interact with this knowledge store to aid appropriate retrieval of information and to guide our behaviour. Despite the importance of semantic cognition in everyday life, there is limited understanding of how these elements of the semantic system change as we age.

Although some prior work has investigated effects of ageing on semantic processing, the vast majority has focused on verbal semantic tasks. However, the multimodal character of semantic cognition, which allows us to translate the meanings of concepts between different modalities (e.g., words and pictures) (Jefferies, 2013; Krieger-Redwood et al., 2015; Patterson et al., 2007), is usually overlooked in cognitive ageing studies. For example, we can retrieve all aspects of information of the concept “headphones,” including its visual properties (overall shape, wire, and buttons), associated actions (put on, take off), and sounds (music), no matter whether we see a picture of headphones or read the word “headphones.” This multimodal nature of semantic processing raises the possibility that information provided by different input modalities may tax semantic cognition systems differently.

On the one hand, fundamental differences in the nature of the information conveyed in different modalities can lead to different types of knowledge representation and activation (Bi, 2021). Specifically, verbal stimuli (e.g., written words) are more related to symbolic, language-derived knowledge representations abstracted away from sensory motor experience, as the forms of words (e.g., being written in one font or another) are generally unrelated to the perceptual properties of their referents. In contrast, non-verbal stimuli (e.g., pictures) tend to elicit more embodied, experience-derived knowledge representations because perceptual properties are inherent in the stimulus itself. In addition, differences in the information provided by different input modalities might also place different executive demands on the semantic system (Krieger-Redwood et al., 2015). For instance, words have more flexible and context-dependent meanings. In contrast, pictures supply a rich array of visual features to engage semantic processing, some of which could be irrelevant to the given task and thus increase the need for inhibition of irrelevant semantic information. These potential differences in processing demands mean that it is essential to take modality into account when investigating age-related shifts in the cognitive architecture of semantics.

It has been established that for healthy individuals, verbal semantic knowledge accumulates throughout the lifespan and is relatively preserved in old age (Grady, 2012; Kavé & Halamish, 2015; Kavé & Yafé, 2014; Park et al., 2002; Salthouse, 2004; Verhaeghen, 2003). These studies have typically indexed semantic knowledge with participants’ scores on vocabulary tests. For example, the Mill Hill vocabulary scale (Raven et al., 1989) asks participants to select synonyms for written words from several alternatives; and the WAIS battery vocabulary subtest (Wechsler, 2008) requires participants to produce verbal definitions of words. However, the non-verbal representation of knowledge has rarely been probed and therefore, little is known about whether non-verbal knowledge follows a similar lifespan trajectory as verbal knowledge.

The contrast of verbal versus non-verbal semantic knowledge has been a greater focus in the neuropsychological literature. In particular, studies of semantic dementia have implicated the bilateral anterior temporal lobes (ATLs) as a “semantic hub” for knowledge representation across all modalities and for all semantic domains (Lambon Ralph & Patterson, 2008; Patterson et al., 2007), and semantic dementia patients with ATL atrophy exhibit a progressive multimodal impairment of both verbal and non-verbal conceptual knowledge (Bozeat et al., 2003; Bozeat et al., 2000; Hodges et al., 1995; Lambon Ralph et al., 2010; Warrington, 1975). This evidence suggests a single store for different types of knowledge. However, other models refute the hypothesis of a unitary semantic store and predict that pictorial and verbal representations are supported separately by the right and left ATLs (Gainotti, 2012, 2014). This argument is consistent with some findings in patients with asymmetric brain damage, which suggest that predominant left ATL atrophy leads to greater damage to verbal knowledge and right ATL atrophy to the loss of non-verbal representations (Butler et al., 2009; Snowden et al., 2004). Other recent proposals suggest a graded specialisation within the two ATLs, with the left showing some specialisation for verbally mediated knowledge and the right for non-verbal (Binney et al., 2012; Lambon Ralph et al., 2017; Rice, Hoffman, & Lambon Ralph, 2015; Rice, Lambon Ralph, & Hoffman, 2015).

In addition to debate over the ATL hub, verbal and non-verbal knowledge might also be processed differently in downstream sensory motor areas. For example, damage to occipitotemporal cortex can result in difficulty recognising a variety of visually presented objects, even when low-level visual perception remains intact and patients can recognise objects when given a verbal definition (Benson & Greenberg, 1969; Farah, 2004; Rubens & Benson, 1971). These results suggest that some regions play a particular role in accessing semantic information from visual inputs. Taken together, these theoretical models and experimental findings indicate that verbal and non-verbal knowledge are supported by the semantic system in different ways, at least to some degree. Thus, when assessing the effects of ageing on semantic cognition, one cannot necessarily generalise the results of verbal semantic knowledge tests to the non-verbal domain.

Compared to the semantic knowledge component, much less is known about how semantic control changes with age. It has commonly been assumed that semantic cognition is preserved in later life because older people have greater reserves of verbal knowledge (Grady, 2012; Kavé & Halamish, 2015; Kavé & Yafé, 2014; Park et al., 2002; Salthouse, 2004; Verhaeghen, 2003). However, age-related changes in semantic control ability have only recently begun to be investigated. Recent studies have shown that the ability to resolve competition between activated knowledge representations (i.e., semantic selection) declines with age, in line with older people’s more general deficits in inhibition; in contrast, controlled retrieval, the ability to probe semantic knowledge for less salient but task-relevant information, is preserved (Hoffman, 2018, 2019; Hoffman & MacPherson, 2022; Wu & Hoffman, 2022; but see Krieger-Redwood et al., 2019). Nevertheless, the evidence so far is limited and these findings are restricted to verbal semantic tasks. We do not know how semantic control changes across adulthood in non-verbal semantic tasks.

Just as with knowledge representation, there is some evidence for neural specialisation for verbal versus non-verbal semantic control. Semantic control processes are supported by the interaction of inferior frontal gyrus (IFG) and left posterior middle temporal gyrus (pMTG) (Jefferies & Lambon Ralph, 2006; Noonan et al., 2013; Whitney et al., 2011). In a previous functional magnetic resonance imaging (fMRI) study with healthy young participants, researchers found that verbal and non-verbal semantic control tasks engaged these semantic control areas differently (Krieger-Redwood et al., 2015). Using word and picture semantic association tasks, the researchers revealed that left posterior IFG, anterior IFG, and left pMTG contributed more to verbal semantic decisions, while right posterior IFG was more activated to picture decisions. These effects suggest that verbal and non-verbal stimuli taxed the semantic control system differently, suggesting that the ageing effects found in verbal semantic control tasks may not generalise to non-verbal tasks.

To provide a clearer understanding of age effects in semantic cognition, the present pre-registered study was designed to investigate how the two key components of semantic cognition change as a function of healthy ageing across verbal (i.e., written word) and non-verbal (i.e., picture) input modalities. We administered a synonym judgement task and a word-to-picture matching task to probe the breadth of verbal and non-verbal semantic knowledge in young and older people. We assessed semantic control ability with picture and word versions of a semantic association task requiring access to weakly related concepts. We expected to replicate the established finding that older people have larger and richer repositories of verbal semantic knowledge. Critically, however, we were also able to test the status of non-verbal semantic knowledge and semantic control abilities in old age.

## Materials and methods

Our sample size, hypothesis, study design, and analyses were pre-registered (available at https://osf.io/936um). The raw data, stimuli, and statistical analysis are also publicly available (https://osf.io/nkeqw).

### Participants

Overall, 51 older adults and 48 young adults were recruited from the Psychology department’s volunteer panel and from the student population, and participated in the study for payment. As a general cognitive screen, the older participants completed the Mini-Addenbrooke’s Cognitive Examination (M-ACE; Hsieh et al., 2015) prior to starting the experiment. Five older participants scored <26 of 30 on the M-ACE, and their data were excluded from the study based on our pre-registered exclusion criteria. One young participant’s data were also excluded because of extremely low accuracy on one of the semantic tasks. Thus, data from 46 older participants (26 females; *M*_age_ = 72.59 years, *SD* = 4.63 years, range = 65–84 years) and 47 young participants (28 females; *M*_age_ = 25.79 years, *SD* = 4.34 years, range = 19–35 years) were included in the analyses. Levels of formal education were high in both age groups (older adults: *M* = 15.89 years, *SD* = 2.64 years, range = 10–20 years; young adults: *M* = 17.45 years, *SD* = 2.21 years, range = 10–23 years), but young adults had completed more years of education than older adults (*t*_91_ = 3.08, two-tailed *p* < .01). This may reflect greater access to higher education in younger generations. All participants were native speakers of English and reported to be in good health with no history of neurological or psychiatric illness. It is worth noting that although all the older participants were British nationals, 11 young participants were from other English-speaking countries (i.e., the United States and Canada). We found that excluding the data from the non-British young people produced qualitatively similar results to including them. Thus, in this article, we report analyses with the data from all qualified participants. Informed consent was obtained from all participants and the research was performed in accordance with all relevant guidelines. The study was approved by the University of Edinburgh Psychology Research Ethics Committee.

### Materials

**Figure 1.**
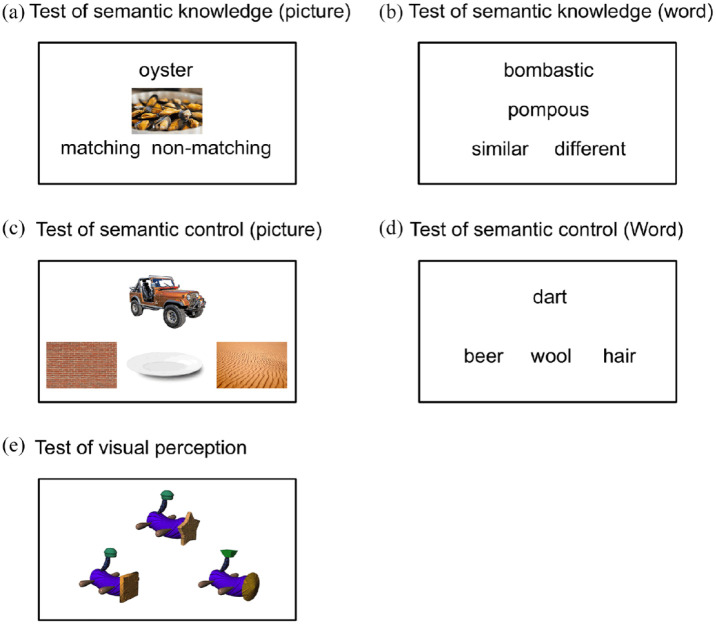
Example items from (a, b) the semantic knowledge tasks, (c, d) the semantic control tasks, and (e) the visual perception task in the experiment.

Three types of tests were completed by the participants, including a semantic knowledge test, a semantic control test, and a visual perception test. Each semantic test had two versions (i.e., picture and word) with different trials (see Figure 1 for examples).

#### Test of breadth of semantic knowledge (picture)

An 80-item word-to-picture matching task was used to probe visual semantic knowledge. On each trial, participants were presented with a probe word and a picture, and were asked to decide whether the object in the picture matched the probe word. On 40 trials, the picture depicted the same concept as the word; on the remaining 40 trials, the word referred to a closely related member of the same semantic category as the pictured object (e.g., oyster—mussel). Thus, the task required people to retrieve specific knowledge of the visual properties of the concept and decide whether these matched the visual features in the picture. To minimise demands on semantic control introduced by the competition between multiple choices, we only included one concept probe and target on each trial (i.e., one word and one picture). The concepts in this task were adapted from the Levels of Familiarity, Typicality, and Specificity (LOFTS) semantic battery (Rogers et al., 2015).

#### Test of breadth of semantic knowledge (word)

Participants completed an 80-item synonym judgement task designed to probe the size of their store of verbal semantic knowledge. On each trial, participants were presented with a pair of words and asked to decide if the two words shared a similar meaning or not. The stimuli for this synonym judgement task were obtained from the norms of the study by Wu and Hoffman (2022). The original norms contain stimuli with parametrically varied difficulty levels, and in the current study, we used the trials at the higher difficulty levels to avoid ceiling effects. This task included some unusual words that were expected to be unknown to some members of the population (e.g., bombastic—pompous). It therefore indexed the breadth of semantic knowledge available to each individual. In total, 40 trials featured synonymous words and 40 unrelated words.

#### Test of semantic control (picture)

An 80-item semantic association task was used to probe the visual semantic control ability of the participants. On each trial, participants were presented with a probe picture along with three option pictures (one target, two foils) and asked to select the probe’s semantic associate. The stimuli in this task were adapted from the study by Krieger-Redwood et al. (2015). We used the trials where the semantic association was weak, in which the automatic activation of semantic knowledge would be insufficient to identify the correct option and participants would instead need to engage in controlled retrieval of semantic information. For example, a participant might be asked whether *jeep* is associated with *wall, dish*, or *desert*. In this situation, processing of the cue may automatically activate the strongest associates of a jeep (wheels, garage, etc.) but fail to relate to any of the available response options. Under these circumstances, individuals are assumed to engage in goal-directed controlled retrieval to search for the relevant information in their semantic store (Badre & Wagner, 2007).

#### Test of semantic control (word)

Similar to the picture semantic control task, an 80-item semantic association task (word version) was used to probe the verbal semantic control ability of the participants. On each trial, participants were presented with a probe word along with three option words (one target, two foils) and asked to select the probe’s semantic associate. The stimuli in this task were also adapted from the study by Krieger-Redwood et al. (2015) and only weakly associated trials were used. The adapted trials in the two semantic control tasks are a subset of the trials used in the original study. The trials selected for use in this study were chosen based on the ratings gathered from an independent set of participants from the study by Krieger-Redwood et al. (2015). The ratings gathered measured two metrics, reaction time and participant judgement on difficulty identifying the correct semantic associate. Participants were presented with a trial, made their judgement, and then were given the correct answer and asked to rate “How easy is it to work out the semantic relationship and reject the distractors?” on a 1 (*not very easy*) to 5 (*very easy*) scale. We selected stimuli to ensure the rated difficulty in the picture and word semantic control tasks was matched (*M*_picture_ = 3.83, *M*_word_ = 3.72, *t*_158_ = 1.09, two-tailed *p* = .28). We used different trials in our word and picture association tasks (i.e., with different probes).

#### Test of visual perception

We used an 80-item novel object matching task as a measure of the participants’ basic visual perception ability. On each trial, participants were presented with one probe picture and two option pictures, and were asked to select the option that shared the greatest visual similarities with the probe object. The stimuli in this novel object matching task were taken from the set of objects known as Fribbles (Tarr, 2003). The Fribbles are a set of 3D novel objects made up of several components (see Figure 1 for example). In the current study, we manipulated the number of shared components between probe, target, and distractor objects in each trial to generate our stimuli. The target always shared three features with the probe while the distractor only shared one or two. Thus, participants needed to resolve competition between targets and distractors based on their visual features but without engaging semantic cognition. We used scores on this task to control for age differences in basic visual perception.

### Procedure

Participants completed the M-ACE followed by the main experiment. The main tasks were presented on a PC running Psychopy software (https://www.psychopy.org/). Participants first completed the two semantic knowledge tasks, then the visual perception task and then the two semantic control tasks. The order of the trials shown in each task was randomised for each participant. Each task began with a set of instructions and a series of practice trials. Each trial began with a fixation cross presented for 1 s, followed by the stimulus pictures/words. In the semantic knowledge tasks, the paired words (or a word and a picture) appeared around the centre of the screen vertically with two options (i.e., similar or different for the word task, and matching or non-matching for the picture task) in a line below (see Figure 1). In the semantic control and the visual perception tasks, the probe picture/word appeared above the centre of the screen with the option pictures/words in a line below. The position of the target was balanced in each task, so that each option had the same chance of being the correct answer. Participants indicated their choice by button press, and their response accuracy and reaction times (RTs) were recorded. Participants were instructed to respond as quickly as possible while avoiding mistakes. They were encouraged to guess if unsure of the correct response. A 5-s time limit was placed on responses for the semantic tasks and a 10-s time limit was placed for trials in the visual perception task.

### Statistical analyses

We first correlated older people’s age with their performance (i.e., accuracy) in our tasks, as a description of the relationship between age in later adulthood and general task processing abilities. Next, before analysing data in the semantic tasks, accuracy and RT on the trials in the visual perception task were first analysed to examine age-related differences in basic visual perception ability. Specifically, we used mixed effects models to predict accuracy and RT at the level of individual trials, with age group as a between-subjects factor. As our results revealed age group differences in visual perception ability, we included visual perception performance (accuracy) in the models for the semantic tasks as a covariate of no interest, to control for the potential influence of basic visual perception ability on our results.

For the semantic tasks, 2 × 2 × 2 factorial mixed models for accuracy and RT were first specified, including age group (older vs young) as a between-subjects factor and modality (picture vs word) and task type (semantic knowledge vs semantic control) as within-subject factors. To investigate the life span changes in semantic knowledge representation and semantic control ability independently, the two components of semantic cognition were also analysed separately in 2 × 2 (age group × modality) models. Mixed effects models were constructed and tested using the recommendations of the study by Barr et al. (2013). Linear models were specified for analyses of RT and logistic models for accuracy. We specified a maximal random effects structure for all models, including random intercepts for participants and task items as well as random slopes for all predictors that varied within-participant or within-item. The age group, modality, and task type were included in the relevant models as categorical predictors. For the accuracy models, we assessed the statistical significance of effects of interest by comparing the full model with a reduced model, which was identical in all respects except for the exclusion of the effect of interest. Likelihood-ratio tests were used to determine whether the inclusion of the effect of interest significantly improved the model fit. For the RT models, the statistical significance of effects of interest was assessed using Satterthwaite’s method with the lmerTest package (Kuznetsova et al., 2017). It is worth noting that, as a reviewer suggested that education level could influence cognitive ability in ageing, we replicated our analyses with the same mixed models but including years of education as an additional covariate. The parallel analyses produced qualitatively similar results, thus we report the results of the models without education level to be consistent with our pre-registered methods.

## Results

### General performance in later adulthood

Before conducting our pre-registered analyses, we first calculated Pearson correlation between age and task accuracy for the older people. We found that age was significantly or marginally significantly correlated with performance in the picture tasks (control picture: *r* = −.42, *p* < .005; knowledge picture: *r* = −.26, *p* = .08; perception: *r* = −.33, *p* < .05), but not the word tasks (control word: *r* = −.23, *p* = .13; knowledge word: *r* = −.18, *p* = .23). This pattern indicates that there is a general age-related decline in tasks involving visual perception in later adulthood, which warrants a further investigation on visual perception ability across age groups and indicates the need to control visual perception performance in the analyses of semantic tasks.

### Visual perception performance of participants

**Figure 2.**
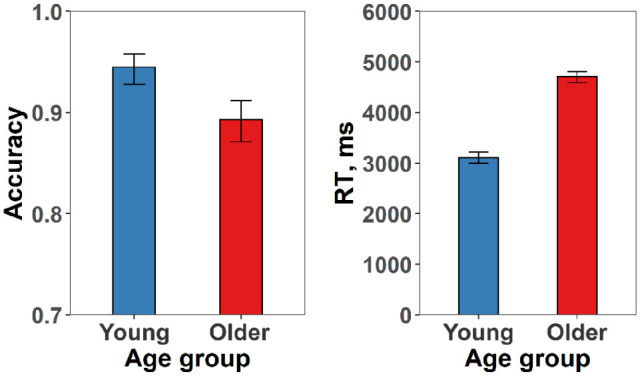
Modelled effects of age group on accuracy and RT in the visual perception task. Error bars indicate 95% confidence intervals.

As shown in Figure 2, we found that older and young adults differed in their basic visual perception ability. Older people were significantly less accurate (*B* = −.359, *SE* = 0.053, *p* < 10^−8^) and slower (*B* = .090, *SE* = 0.003, *p* < 10^−15^) than young people in the visual perception task. To exclude the potential influence of individual differences in basic visual perception on our results, we included each participant’s response accuracy for the visual perception task in the semantic tasks mixed effects models as a covariate of no interest. We used accuracy as the covariate, as the age-related RT difference was likely due to variations in general processing speed that were not specific to a particular cognitive domain (Salthouse, 1996).

### Effects of age and modality on semantic knowledge and control

The accuracy and RT data on the semantic tasks were first analysed in a 2 × 2 × 2 factorial mixed model that included age group (older vs young) as a between-subjects factor and task type (semantic knowledge vs semantic control) and modality (picture vs word) as within-subject factors. The results are shown in Table 1. Overall, older people produced significantly more accurate but slower responses than young people. There was also a significant main effect of modality for accuracy, and main effects of modality and task type for RT. That is, people were generally slower and less accurate for pictures and they were slower (but not less accurate) on the semantic control tasks. Importantly, there were significant three-way and two-way interactions between these factors, necessitating post hoc tests of the effects in each task. It is worth noting that, the covariate of visual perception performance had a significant effect on the accuracy model, indicating that the high performers in the visual perception task were also more accurate in the semantic tasks.

**Table 1.** Mixed effects models predicting accuracies and RTs in the semantic tasks from age group, modality, and task type.

	Accuracy			RT		
Effect	*B*	*SE*	*p*	*B*	*SE*	*p*
Age group	.158	0.048	<.001	.041	0.007	<10^−7^
Task type	−.016	0.099	.782	.074	0.006	<10^−15^
Modality	−.416	0.099	<10^−4^	.010	0.005	<.05
Visual perception	.118	0.034	<.001	−.0001	0.006	.990
Age group × task type	−.178	0.041	<10^−5^	.008	0.003	<.05
Age group × modality	−.116	0.041	<.01	.017	0.003	<10^−9^
Task type × modality	.251	0.099	<.01	.004	0.005	.465
Age group × task type × modality	−.032	0.040	.444	.005	0.002	<.05

RT: reaction times.

**Figure 3.**
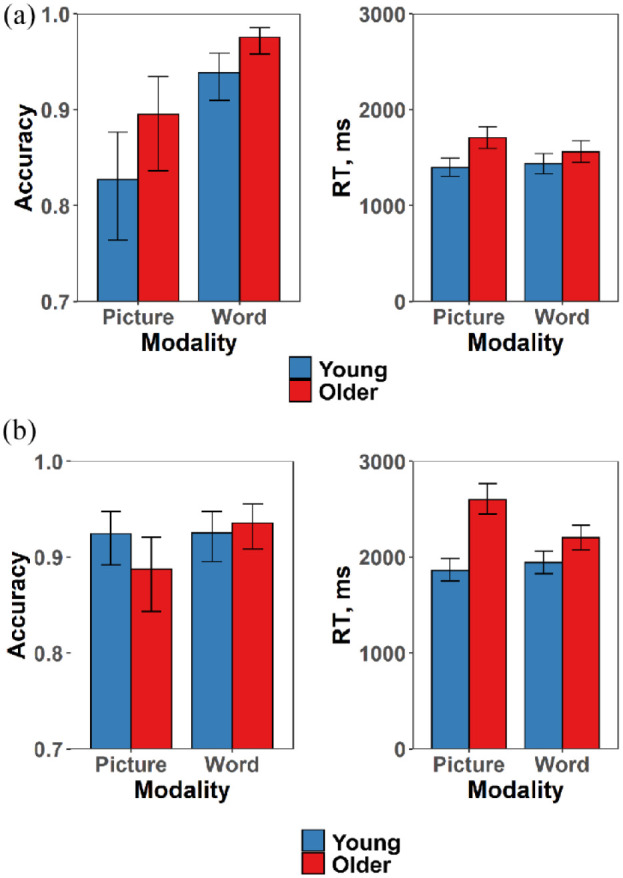
Modelled effects of age group and modality on accuracy and RT in (a) the semantic knowledge tasks and (b) the semantic control tasks. Error bars indicate 95% confidence intervals.

The nature of the interactions was investigated with separate 2 × 2 (age group × modality) analyses for each type of semantic task. The parameter estimates for the accuracy and RT models are presented in Table 2, and the modelled effects of the predictors are shown in Figure 3. For the knowledge-based tasks, there were main effects of age group and modality for accuracy but the effect of modality did not interact with age group. In other words, older people were more accurate in the semantic knowledge tasks irrespective of the input modality, indicating that older people’s superiority in verbal semantic knowledge also extends to picture-based knowledge. For RT, there was, again, a main effect of age group (older people were slower overall) and this interacted with modality, as older adults were particularly slow in the picture condition.

**Table 2.** Mixed effects models predicting accuracies and RTs in the semantic knowledge and control tasks from age group and modality.

Effect	Accuracy			RT		
	*B*	*SE*	*p*	*B*	*SE*	*p*
Semantic knowledge task						
Age group	.381	0.073	<10^−7^	.031	0.008	<.001
Modality	−.673	0.155	<10^−5^	.007	0.008	.381
Visual perception	.103	0.051	<.05	−.005	0.008	.529
Age group × modality	−.092	0.067	.107	.013	0.004	<.01
Semantic control task						
Age group	−.068	0.056	.257	.050	0.007	<10^−9^
Modality	−.157	0.127	.209	.014	0.006	<.05
Visual perception	.109	0.042	<.05	.002	0.007	.737
Age group × modality	−.148	0.046	<.01	.022	0.003	<10^−10^

RT: reaction times.

For the semantic control tasks, there was no overall difference in accuracy between young and older people or between picture and word modalities. There was, however, an age group × modality interaction, which indicated that older people made more errors than the young in the picture condition, even though their performance was similar to young people in the word condition. Analysis of RTs showed that older people were slower overall in the semantic control tasks and that there was, again, an age group × modality interaction, as older people were particularly slow in the picture condition. Thus, when required to identify weak semantic associations between concepts, older people showed a disproportionate deficit for picture stimuli, compared with words.

## Discussion

This study investigated age-related changes in semantic knowledge representation and semantic control ability across different input modalities. Previous research has examined age-related changes in semantic cognition using verbal stimuli exclusively, thus little is known about these capabilities when older people are faced with non-verbal inputs. Given that the literature has revealed neural dissociations between verbal and non-verbal knowledge representations and semantic control processes, the patterns of age-related changes to semantic cognition might vary based on input modality. This study provides novel and important data in advancing our understanding of this question. We found evidence for two distinct patterns of change across adulthood for semantic knowledge and semantic control processing. Performance on tests of both verbal and non-verbal knowledge representations showed a unanimous improvement in older age. In contrast, while verbal controlled-retrieval ability was maintained in older people, there was a clear decrement in the non-verbal condition, which was not explained by basic visual perception ability. Taken together, these findings reveal a full picture of the interaction between input modality, healthy ageing, and different components of semantic cognition.

Previous investigations have suggested a semantic knowledge increase during adulthood, as older people often perform better than the young on tests that probe vocabulary size (Grady, 2012; Kavé & Halamish, 2015; Kavé & Yafé, 2014; Park et al., 2002; Salthouse, 2004; Verhaeghen, 2003). Consistent with these findings, this study found that the older group outperformed young people in accuracy in the verbal semantic knowledge task. More importantly, we also found a similar age-related advantage in the picture knowledge task. These findings clearly indicate that older people have a more detailed store of knowledge representations that allows them to more successfully understand the meanings of concepts obtained from both verbal and non-verbal input channels. While older adults were generally more accurate than their younger counterparts across modalities on the knowledge tasks, they took longer to make their decisions, which might reflect a general slower response speed in older age that is not specific to semantics (Salthouse, 1996). Furthermore, while older adults were consistently more accurate, they were particularly slow in the picture condition. This may indicate less efficient retrieval of semantic information from images in older age, as we will discuss later. Overall, however, the knowledge tasks imply that verbal and non-verbal knowledge continue to develop in parallel throughout adulthood.

The semantic association tasks, which required participants to retrieve less dominant information from their semantic store, were used to probe participants’ semantic control ability. Previous work suggested that semantic control consists of semantic-specific elements, which regulate how information is retrieved from the semantic store, and domain-general elements, which are involved in resolving competition between active representations (Badre et al., 2005; Hoffman, 2018; Nagel et al., 2008). The current study used semantic association tasks, which tapped the former component, as a measure of participants’ semantic control ability. In line with previous findings (Hoffman, 2018, 2019; Hoffman & MacPherson, 2022; Wu & Hoffman, 2022), we found that the older participants were as effective as young people when retrieving weak associations based on verbal inputs. Interestingly, this was not the case for the picture association task. Here, older adults showed a decline in both response accuracy and speed in comparison with the young, even though performance in basic visual perception was controlled for.

There are two potential explanations for the interaction between age and modality in the semantic control tasks. The first is that differences in the information provided by word and picture stimuli place different executive demands on the semantic system, to the detriment of older people. Unlike words, pictures explicitly provide a rich set of visual features, which lead to automatic activation of a large set of semantic features for the depicted objects. Many of these features will not be useful for identifying the relevant association. For example, when processing a picture of a jeep (as in Figure 1c), participants may automatically activate information about its headlights, windows, and mirrors, and semantically irrelevant properties, such as its colour. These properties must be ignored if participants are to focus on the deeper knowledge that this type of vehicle is suited to use in the desert. Thus, picture-based association tasks may incur extra semantic selection and inhibition demands that are not present in word-based tasks. Previous findings indicate that older people have particular deficits inhibiting irrelevant semantic information, and in a range of non-semantic tasks requiring interference resolution and inhibition (Borella et al., 2008; Hasher & Zacks, 1988; Hoffman, 2018; Salthouse & Meinz, 1995; Wu & Hoffman, 2022). Thus, the increased inhibitory requirements in the picture association task could lead to impaired performance in older people.

An alternative possibility is that the older people were less efficient at accessing semantic information from pictures, which slowed them down and caused more errors. Researchers have found that the ventral occipital-temporal cortex, which contains clusters responding selectively to different object domains (e.g., faces, places, and tools) and is essential for visual recognition, becomes less functionally specialised with age (Park et al., 2004). This age-related neural dedifferentiation for object domains might affect older people’s ability to recognise object pictures (and to differentiate relevant from irrelevant information) and lead to worse performance in the picture association task (Rémy et al., 2013). Although the word-to-picture matching task (the picture knowledge task) also involved visual object recognition, this task used only one picture in each trial (cf. four pictures per trial in the association task) so might be less influenced by the participants’ object recognition ability. It is worth noting that, although our analyses controlled participants’ basic visual perception ability using their performance on the novel object matching task, higher-level visual recognition ability supported by the ventral visual pathway (which is also part of the semantic system) was not measured in the current study (Bi et al., 2016; Downing et al., 2005; Pietrini et al., 2004). It is important for future confirmatory studies to investigate in more depth how the cognitive and neural mechanisms of object recognition change as a function of age.

In conclusion, this study manipulated tasks tapping different aspects of semantic cognition and input modalities to produce a full picture of age-related changes in semantic abilities across verbal and non-verbal modalities. We found that while older people achieved better performance in both the verbal and non-verbal semantic knowledge tasks, their non-verbal semantic control ability declined in old age in comparison with verbal semantic control. These changes were independent of the basic visual perception ability of the individuals. Our study has not only revealed the shifting cognitive architecture of semantics in later life but has also highlighted the importance of taking input modality into account in cognitive ageing studies in other domains.
